# Effect of Co-culturing Fibroblasts in Human Skeletal Muscle Cell Sheet on Angiogenic Cytokine Balance and Angiogenesis

**DOI:** 10.3389/fbioe.2020.578140

**Published:** 2020-09-23

**Authors:** Parichut Thummarati, Masahiro Kino-oka

**Affiliations:** Department of Biotechnology, Graduate School of Engineering, Osaka University, Osaka, Japan

**Keywords:** skeletal muscle cell sheet, fibroblast co-culture, cytokine balance, myoblast alignment, angiogenesis, tissue engineering

## Abstract

Skeletal muscle comprises a heterogeneous population of myoblasts and fibroblasts. Autologous skeletal muscle myoblasts are transplanted to patients with ischemia to promote cardiac regeneration. In damaged hearts, various cytokines secreted from the skeletal muscle myoblasts promote angiogenesis and consequently the recovery of cardiac functions. However, the effect of skeletal muscle fibroblasts co-cultured with skeletal muscle myoblasts on angiogenic cytokine production and angiogenesis has not been fully understood. To investigate these effects, production of vascular endothelial growth factor (VEGF) and hepatocyte growth factor (HGF) was measured using the culture medium of monolayers prepared from various cell densities (mono-culture) and proportions (co-culture) of human skeletal muscle myoblasts (HSMMs) and human skeletal muscle fibroblasts (HSMFs). HSMM and HSMF mono-cultures produced VEGF, whereas HSMF mono-culture produced HGF. The VEGF productivity observed in a monolayer comprising low proportion of HSMFs was two-fold greater than that of HSMM and HSMF mono-cultures. The production of VEGF in HSMMs but not in HSMFs was directly proportional to the cell density. VEGF productivity in non-confluent cells with low cell-to-cell contact was higher than that in confluent cells with high cell-to-cell contact. The dynamic migration of cells in a monolayer was examined to analyze the effect of HSMFs on myoblast-to-myoblast contact. The random and rapid migration of HSMFs affected the directional migration of surrounding HSMMs, which disrupted the myoblast alignment. The effect of heterogeneous populations of skeletal muscle cells on angiogenesis was evaluated using human umbilical vein endothelial cells (HUVECs) incubated with fabricated multilayer HSMM sheets comprising various proportions of HSMFs. Co-culturing HSMFs in HSMM sheet at suitable ratio (30 or 40%) enhances endothelial network formation. These findings indicate the role of HSMFs in maintaining cytokine balance and consequently promoting angiogenesis in the skeletal muscle cell sheets. This approach can be used to improve transplantation efficiency of engineered tissues.

## Introduction

Transplantation of skeletal muscle myoblasts is a promising therapeutic strategy for myocardial infarction (Pagani et al., 2003). Easy availability and lack of immunologic barriers are the major advantages of using skeletal muscle myoblasts for transplantation (Dib et al., 2005b; Menasche, 2007). Furthermore, transplantation of autologous skeletal muscle myoblasts into the heart is reported to be safe and efficient in humans (Menasche et al., 2001; Durrani et al., 2010). Previous studies have revealed that various factors of skeletal muscle myoblasts induce angiogenesis and recruit the progenitors at the infarcted area, which result in the induction of cardiac tissue and recovery of heart function (Suzuki et al., 2001; Murtuza et al., 2004; Memon et al., 2005; Shirasaka et al., 2013). Additionally, the implantation of multilayered skeletal muscle myoblast sheets enhances angiogenesis both *in vitro* (Ngo et al., 2013) and *in vivo* (Sekiya et al., 2009; Miyagawa et al., 2017).

Similar to myoblasts, fibroblasts, which are the most common cell type in the connective tissues, can synthesize and secrete proangiogenic growth factors such as vascular endothelial growth factor (VEGF) and hepatocyte growth factor (HGF). In addition, fibroblasts synthesize extracellular matrix (ECM) components, such as collagen, fibronectin and proteoglycans that can promote angiogenesis in ischemia areas (Newman et al., 2011; Kendall and Feghali-Bostwick, 2014; Chapman et al., 2016). However, increased number of fibroblasts may result in excessive deposition of ECM and consequently fibrosis (Mann et al., 2011; Kendall and Feghali-Bostwick, 2014). Thus, co-transplantation of skeletal muscle myoblasts and a small proportion of fibroblasts can be a potential strategy for myocardial tissue regeneration. The proportion of fibroblasts and myoblasts in the skeletal tissue may vary depending on the tissue source, which may affect the therapeutic efficacy of transplantation. There is limited understanding of the effect of heterogeneous populations of skeletal muscle myoblasts and fibroblasts on cytokine production and angiogenesis.

Various potent growth factors are reported to function as angiogenic simulators in ischemic areas. VEGF, HGF, and basic fibroblast growth factor (bFGF or FGF2), which are direct proangiogenic markers that promote angiogenesis (Fallah et al., 2019; Laddha and Kulkarni, 2019), are experimentally demonstrated to improve cardiac functions. Combined delivery of HGF and VEGF to infarcted myocardium showed an increase of left ventricle (LV) wall thickness and capillary density, reduce myocardial infarction size and improve dilatation index (Makarevich et al., 2018). Clinical trials have demonstrated enhancing myocardial perfusion leading to a better cardiac function and well-tolerated following therapy with VEGF, HGF, and FGF2 (Atluri and Woo, 2008). VEGF exerts its physiological functions by binding to two homologous VEGF receptors, which are expressed on vascular endothelial cells (Carmeliet, 2005; Fallah et al., 2019). VEGF directly acts on the endothelial cells to enhance migration, increase permeability, and enhance survival during vascularization and angiogenesis (Zachary and Gliki, 2001). Injection of skeletal myoblasts with genetic modifications to upregulate the expression of VEGF was reported to effectively treat acute myocardial infarction through vasodilatory and angiogenic effects (Suzuki et al., 2001; Haider et al., 2004). However, this therapeutic strategy of gene transfer involves viral vectors, which are associated with adverse effects and ethical concerns (Kim et al., 2001). HGF, a potent mitogen for various cell types, including endothelial cells, promotes endothelial cell motility, interaction, branching morphogenesis, and/or tubular morphogenesis during angiogenesis and vascularization (Morimoto et al., 1991; Rosen et al., 1997). Furthermore, previous studies have demonstrated the therapeutic effects of HGF on myocardial infarction *in vivo* (Nakamura et al., 2000; Ueda et al., 2001; Jin et al., 2003; Liu et al., 2016). The HGF-engineered skeletal myoblasts promote angiogenesis, reduce myocardial fibrosis, and decrease apoptosis of cardiomyocytes (Yuan et al., 2008; Madonna et al., 2015). FGF2 is also reported to exert therapeutic effects in ischemia by regulating angiogenesis through regulation of various cell-cell interactions (Murakami and Simons, 2008) and other growth factors or chemokines, including VEGF (Masaki et al., 2002; Kanda et al., 2004) and HGF (Onimaru et al., 2002).

This study aimed to investigate the effect of co-coculturing human skeletal muscle fibroblasts (HSMFs) with human skeletal muscle myoblast (HSMM) sheets on cytokine balance and angiogenesis *in vitro*. Angiogenic cytokine productivity was measured in the monolayers prepared from various seeding densities (mono-culture) and proportions (co-culture) of HSMFs and HSMMs. The dynamic behavior of cells in the monolayer was analyzed to understand the mechanism underlying cytokine production in the skeletal muscle cells. The effect of co-culturing HSMFs in five-layered HSMM sheet on angiogenesis was evaluated using an *in vitro* angiogenesis model mimicking the transplantation area.

## Materials and Methods

### Cell Culture and Preparation

In this study, human skeletal muscle cells (Lot. No. 6F4296; Lonza, Walkersville, Inc., Walkersville, MD, United States) that contain HSMMs and HSMFs were used for the experiments. The skeletal muscle cells were sub-cultured at 37°C and 5% CO_2_ in skeletal cell growth medium-2 (SkGM-2; Lonza, Walkersville, MD, United States), which was formulated by combining SkBM-2 Basal Medium (Cat. No. CC-3246) and SkGM-2 SingleQuots Supplements and Growth Factors (Cat. No. CC-3244). The desmin-positive and desmin-negative cells were identified as HSMMs and HSMFs, respectively (Kino-oka et al., 2013). The proportion of HSMMs and HSMFs in the original human skeletal muscle cells or “pre-sorted cells” was 68.1 and 31.9%, respectively (Supplementary Figure 1).

The HSMMs and HSMFs were sorted using fluorescence-activated cell sorting (FACS). The pre-sorted cells were stained with Alexa Fluor 488-conjugated anti-CD56 (NCAM) antibody (Cat. No. 2191555, Sony, Sony Biotechnology, Inc., San Jose, CA, United States) at 5 μl to 1 × 10^6^ cells, following the manufacturer’s instructions. The cells were then sorted using a cell sorter (JZAN JR; Bay Bioscience, Co., Ltd., Japan) based on cell size and CD56-Alexa Fluor 488 fluorescence intensity (Supplementary Figure 1D). The sorted CD56^+^ and CD56^–^ cells (post-sorted cells) were cultured at 37°C and 5% CO_2_ in SkGM-2 medium. The purity of pre-sorted cells and post-sorted cells was determined by culturing the cells in SkGM-2 medium for 24 h. Next, the cells were fixed with 4% formaldehyde (Wako Pure Chemical Industries, Tokyo, Japan) for 15 min and permeabilized with 0.5% Triton X-100 for 20 min. The cells were blocked with 1% bovine serum albumin (BSA) for 90 min and incubated with a mixture of anti-desmin antibody (Y66) (Cat. No. ab32362, Abcam, United States) at 1:250 dilution, and anti-fibroblast antibody (clone TE-7) (Cat. No. CBL271, Millipore, United States) at 1:100 dilution prepared in 1% (w/v) BSA at 4°C overnight. The cells were washed and immunolabeled with a mixture of Alexa Fluor 594 goat anti-rabbit IgG (Cat. No. A11001, Molecular Probes, Life Technologies, United States) at 1:250 dilution and Alexa Fluor 488 goat anti-mouse IgG (Cat. No. A11012, Molecular Probes, Life Technologies, United States) at 1:250 dilution prepared in 1% (w/v) BSA for 1 h at room temperature, followed by counterstaining with 4′-6-diamidino-2-phenylindole (DAPI) (Cat. No. D1306, Molecular Probes, Life Technologies, United States).

### Culturing HSMMs and HSMFs at Various Initial Cell Densities

The sorted HSMMs or HSMFs were cultured in SkGM-2 medium at various initial seeding densities (*X*_0_) (0.1 × 10^5^, 0.8 × 10^5^, and 3.5 × 10^5^ cells/cm^2^) inside a Teflon ring (area: 0.95 cm^2^) placed in 24 well plates. The Teflon rings were removed after incubation at 37°C and 5% CO_2_ for 24 h. The cultured medium was replaced every 24 h and collected at 72 h for measuring the levels of cytokines.

### Preparation of Monolayer With Various HSMM:HSMF Ratios

The sorted HSMMs were mixed with HSMFs in various proportions from 0 (no HSMF) to 100% HSMF (HSMF:HSMM ratios from 0:20 to 20:0) and seeded inside a Teflon ring (area: 0.95 cm^2^) placed in 24 well plates at *X*_0_ of 3.5 × 10^5^ cells/cm^2^. The cells were incubated for 24 h at 37°C and 5% CO_2_ to form the monolayer. After 24 h, the Teflon rings were removed, and the culture medium was replaced with fresh medium. The cultured medium was collected at 72 h for cytokine measurement.

To prepare a monolayer for cell alignment analysis, the sorted HSMFs were stained with CellTracker Green CMFDA (Cat. No. C7025, Invitrogen, Thermo Fisher Scientific, United States). Next, the stained HSMFs were mixed with HSMMs and seeded (*X*_0_ of 3.5 × 10^5^ cells/cm^2^) inside a Teflon ring (area: 0.95 cm^2^) placed in 24 well plates. After 24 h, the Teflon rings were removed, and the culture medium was replaced with fresh medium. At 48 h, the attached cells were stained with NucBlue Live ReadyProbe Reagent (Hoechst 33342) (Cat. No. R37605, Molecular Probe, Life Technologies, United States), following the manufacturer’s instructions.

### Quantitative Analysis of HSMM and HSMF Migration and Cell Alignment

**FIGURE 1:**
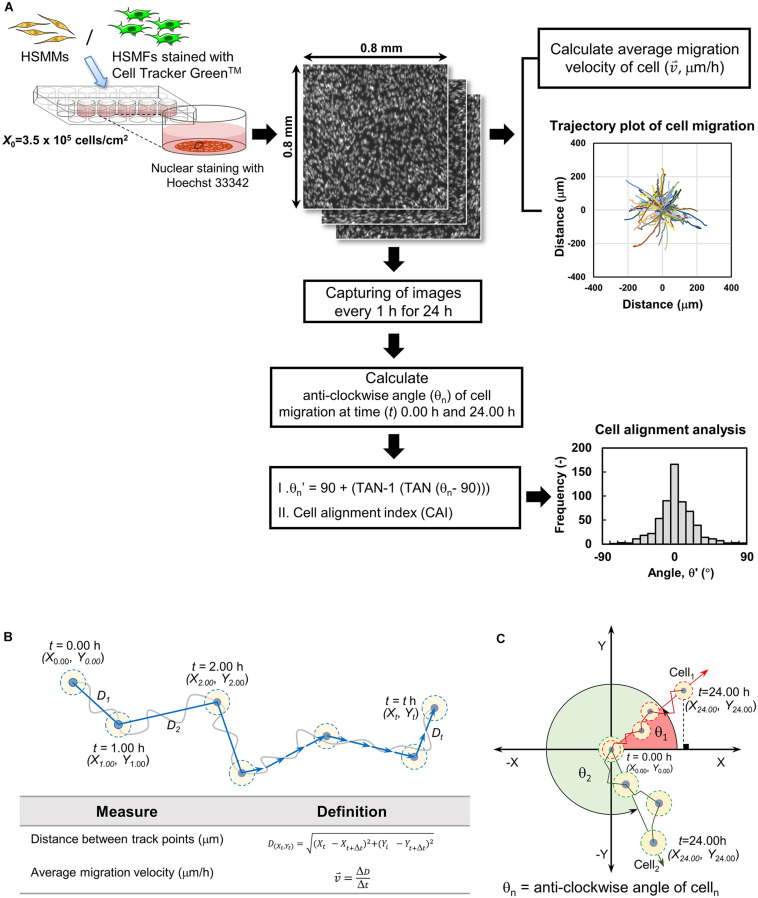
Quantifying cell migration in monolayer using time-lapse image data analysis. **(A)** Diagram showing experimental procedure and data analysis of time-lapse images. The cells in the monolayer prepared using skeletal muscle cells with various proportion of human skeletal muscle fibroblast (HSMF) and human skeletal muscle myoblast (HSMM) were stained with Hoechst 33342. The images were captured every 1 h during 48–72 h of incubation. The positions (X,Y) of the nucleus were measured using Image J with Manual tracking plugin to evaluate the migration velocity (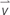, μm/h), direction angle (θ′), and cell alignment index (CAI) for cell alignment analysis. **(B)** Cell positions at each hour were measured to calculate the distance between track point and average migration velocity. **(C)** The positions of the cell at origin (*t* = 0.00 h) and at the end point (*t* = 24.00 h) were measured to evaluate the anti-clockwise angle (θ′) of cell migration.

Figure 1 shows the analysis of cell migration and alignment data from cell tracking and time-lapse observation. Images of stained nuclei and HSMFs in the monolayer were captured every 1 for 24 h using the IN Cell Analyzer 2000 (GE Healthcare) under a 20X objective lens. The original images (1.5 mm × 1.5 mm) of stained cells were captured in an 8-bit gray scale with a resolution of 0.74 μm/pixel. The images were captured from three random areas from duplicate samples in the 24 well plates. For quantitative analysis, 100 nucleus-stained cells from the local density in three regions of interest (ROI; 0.8 mm × 0.8 mm) from duplicated samples were used. The cells from sequence images were measured at position (X,Y) using Image J software (Manual tracking plugin), and the data were visualized in a trajectory plot. The migration velocity (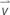, μm/h) and directional angle [θ and θ′, degree (°)] were quantified as shown in Figures 1B,C, respectively. The frequency of migration angle of cells during 48–72 h of incubation was plotted in a histogram. The cell alignment index (CAI) was defined as the amount of variation (standard deviation, σ) of the migration angle of cells. A high σ value indicates a high degree of cell alignment disruption.

### Measurement of Cytokine Productivity

Cytokine levels were measured in the cultured medium collected from triplicate samples at 72 h. The levels of VEGF, HGF, and FGF2 were measured using the human VEGF Quantikine enzyme-linked immunosorbent assay (ELISA) assay (Cat. No. DVE00, R&D Systems, Inc., United States), human HGF ELISA Kit (Cat. No. KAC2211, Invitrogen, Thermo Fisher Scientific, United States), and human FGF Quantikine ELISA (Cat. No. DFB50, R&D Systems, Inc., United States), respectively, following the manufacturer’s instructions. The Sf 21-expressed recombinant human VEGF165, *E. coli*-expressed recombinant human basic FGF and recombinant human HGF provided from the ELISA kits were used to establish the standard curve. The baseline levels of these cytokines in cultured media without cells were measured. At 72 h, the cells in monolayer were fixed with 4% paraformaldehyde and the nuclei were stained with DAPI (Cat. No. D1360, Invitrogen, Thermo Fisher Science, United States) to determine the total cell number in the monolayer. Cytokine productivity refers to the amount of cytokine (pg) produced per cell per hour (pg/cell × h) or per sheet (pg/sheet × day).

### Incubation of Five-Layered HSMM Sheets With Green Fluorescent Protein-Tagged Human Umbilical Vascular Endothelial Cells (GFP-HUVECs)

A five-layered HSMM sheet containing different proportions of HSMFs was fabricated according to a previous method (Nagamori et al., 2013). Briefly, the HSMMs and HSMFs were sub-cultured and mixed at various proportions of 0 (no HSMF), 10, 30, and 40% HSMF (HSMF:HSMM ratios of 0:20, 2:18, 6:14, and 8:12). The cells were seeded at *X*_0_ of 3.5 × 10^5^ cells/cm^2^ inside Teflon rings (0.95 cm^2^) placed in the 24 well UpCell plates (CellSeed, Tokyo, Japan) with a temperature-responsive surface. The cells were incubated for 24 h at 37°C and 5% CO_2_ to allow the formation of a monolayer sheet. To harvest the monolayer sheet, a gelatin stamp was overlaid onto the monolayer sheet in a well at 37°C. The temperature was decreased to 20°C, and the stamp was removed with the monolayer sheet from the bottom surface of the well. These steps were then repeated to sequentially harvest the monolayer sheets to form a multilayered construct. The multilayered sheet was transferred to the center of a 35 mm culture dish (ibidi GmbH, DE), which was seeded with GFP-HUVECs (Lot. No. 20100201001; Angio-Proteomie, MA, United States) (*X*_0_ of 0.1 × 10^5^ cells/cm^2^) in endothelial cell growth media-2 (EGM-2) at 37°C and 5% CO_2_ for 24 h. At the sampling time (*t*), triplicate samples were harvested for quantitative analysis. During the incubation period, the medium was changed every day.

### Evaluation of GFP-HUVEC Network Formed Inside Multilayered HSMM Sheet

The images of eight positions in each sample were captured using a 10X objective lens of a confocal laser scanning microscope (FV-10i, Olympus, Tokyo, Japan). All images were converted to 8-bit gray scale with a size of 256 × 256 pixels covering an area of 1.27 mm × 1.27 mm. The images were processed (Image-Pro Plus; Media Cybernetics, Inc., Bethesda, MD, United States) using a low-pass filter for primary noise removal and binarization with a fixed intensity threshold. The threshold intensity was set as the average of the mode intensity and automatic threshold intensity. The binary images were subjected to skeletonization and secondary noise removal with a size threshold to remove items with a size of less than 16 pixels. The small branches were pruned in the objects. The total length of the network per image area (*L*; cm^–1^) and the number of total tips of the network (*N*_*T*_; tip/cm^2^) were measured to estimate the degree of HUVEC network formation (*L*/*N*; cm/tip). The tips at the edge of the image were not counted.

### Statistical Analysis

All experimental data are expressed as mean ± standard deviation. Each experiment was performed on three independent sample (*n* = 3) for quantitative analysis. The differences among multiple groups were evaluated using one-way analysis of variance (ANOVA), followed by Bonferroni or least-significant different (LSD) *post hoc* test (SPSS 26.0). The differences were considered statistically significant when the *P*-value was less than 0.01.

## Results

### Effect of HSMF on Cytokine Productivity in HSMM Monolayer

**FIGURE 2:**
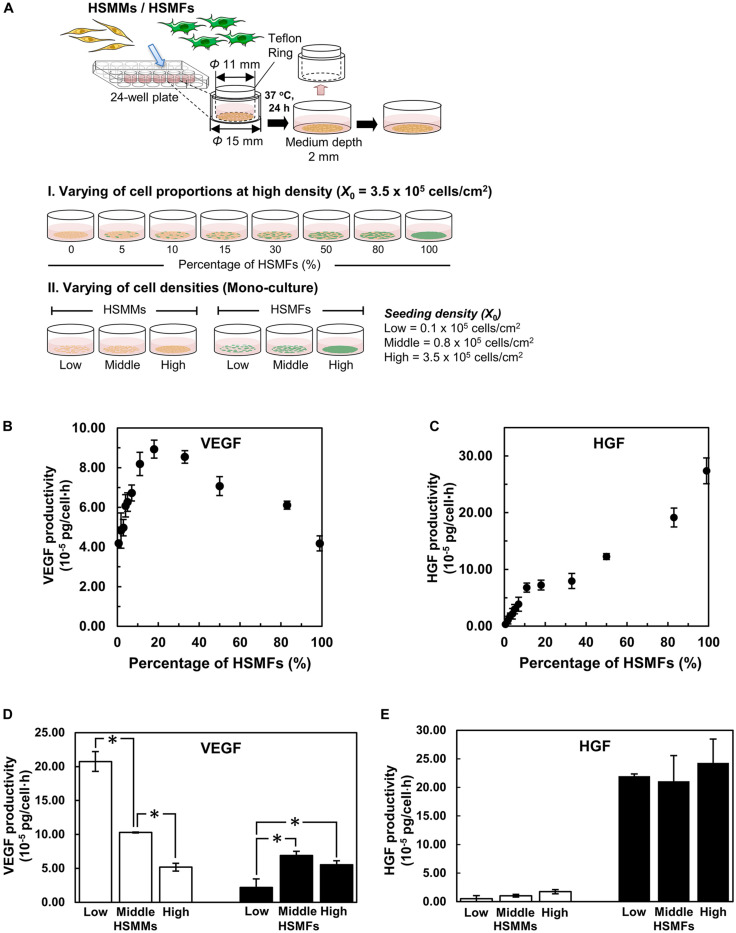
Cytokine productivity in monolayers of skeletal muscle cells. **(A)** A schematic drawing shows the experimental design. The monolayers were prepared with various proportions of HSMFs and HSMMs (I) or using various initial seeding densities (*X*_0_) of HSMFs or HSMMs (II). The culture medium of monolayers was collected at 72 h to measure the cytokine levels using enzyme-linked immunosorbent assay (ELISA). **(B)** Effect of co-culturing HSMFs and HSMMs with various proportions in a high initial density on vascular endothelial growth factor (VEGF) productivity and **(C)** hepatocyte growth factor (HGF) productivity. **(D)** The HSMMs and HSMFs were cultured various *X*_0_ at 0.1 × 10^5^ (low), 0.8 × 10^5^ (middle) and 3.5 × 10^5^ cells/cm^2^ (high) for 72 h. The productivities of vascular endothelial growth factor (VEGF) and hepatocyte growth factor (HGF) **(E)** in the culture medium were measured. Data are represented as average cytokine productivity ± standard deviation from triplicate samples (*n* = 3). *P < 0.01; one-way analysis of variance (ANOVA), followed by Bonferroni *post hoc* test.

Cytokine production in the monolayers prepared from skeletal muscle cells with various proportions of HSMF from no HSMF to 100% HSMF (HSMF:HSMM ratios from 0:20 to 20:0) was investigated after 72 h of culturing. The culture media of different groups were collected at 72 h to measure the levels of VEGF, HGF, and FGF2 (Figure 2A). VEGF productivity in monolayers derived from mono-cultures of HSMMs and HSMFs were 4.19 ± 0.10 × 10^–5^ and 4.18 ± 0.38 × 10^–5^ pg/cell × h, respectively. The VEGF productivity in the monolayer prepared from skeletal muscle cells with 15% (HSMF:HSMM ratio of 3:17) HSMF was approximately twofold higher (8.93 ± 0.45 × 10^–5^ pg/cell × h) than that in the monolayer prepared from mono-cultures of HSMF or HSMM (*P* < 0.01). The VEGF productivity decreased when the percentage of HSMF was higher than 15% (Figure 2B). In contrast to VEGF productivity, HGF productivity in the monolayer derived from HSMM mono-culture was low (0.33 ± 0.33 × 10^–5^ pg/cell × h). HGF productivity in the monolayer derived from HSMF mono-culture was 27.4 ± 2.27 × 10^–5^ pg/cell × h (Figure 2C). The baseline levels of VEGF and HGF in culture media without cells used in this experiment were 0.11 ± 0.19 and 0.05 ± 0.09 pg/ml, respectively. The levels of FGF2 were almost undetectable under all conditions (data not shown). These results indicate that varying the proportions of HSMM and HSMF in the skeletal cell monolayer differentially affected the production of cytokines. HGF was secreted only by HSMFs. The production of VEGF in the HSMM monolayers can be upregulated by introducing a small proportion of HSMFs.

### Effect of Cell Density on Cytokine Productivity in HSMMs and HSMFs

The effect of a small proportion of HSMFs in the HSMM monolayers on VEGF productivity was evaluated by examining the cell-to-cell contact. The productivities of VEGF in the sorted HSMMs cultured at low (0.1 × 10^5^ cells/cm^2^), medium (0.8 × 10^5^ cells/cm^2^), and high (3.5 × 10^5^ cells/cm^2^) *X*_0_ values, which resulted in varying degrees of cell-to-cell contacts, were compared with those of HSMFs. The productivity of VEGF in the monolayer derived from low *X*_0_ value of HSMMs was 20.7 ± 1.46 × 10^–5^ pg/cell × h. However, the productivity of VEGF in the monolayers derived from medium and high *X*_0_ values of HSMMs significantly decreased to 10.3 ± 0.07 × 10^–5^ (*P* < 0.01) and 5.18 ± 0.58 × 10^–5^ pg/cell × h (*P* < 0.01), respectively. The productivity of VEGF in the HSMF mono-culture varied, which was not related to *X*_0_. The productivities of VEGF in the monolayer derived from low, medium, and high *X*_0_ values of HSMFs were 2.18 ± 1.27 × 10^–5^, 6.90 ± 0.61 × 10^–5^, and 5.53 ± 0.60 × 10^–5^ pg/cell × h, respectively (Figure 2D). The productivity of HGF was high in the monolayer derived from HSMF mono-cultures at all *X*_0_ values but was almost undetectable in the monolayer derived from HSMM mono-culture. The productivities of HGF in the monolayers derived from low, medium, and high *X*_0_ values of HSMFs were 22.0 ± 0.39 × 10^–5^, 21.1 ± 4.47 × 10^–5^, and 24.3 ± 4.16 × 10^–5^ pg/cell × h, respectively. Additionally, the productivity of HGF was similar in the monolayers prepared from different *X*_0_ values of HSMFs (Figure 2E). These results suggest that HSMF-mediated myoblast-myoblast contact disruption might promote VEGF production in HSMMs.

### Migration of HSMMs and HSMFs in the Monolayer

**FIGURE 3:**
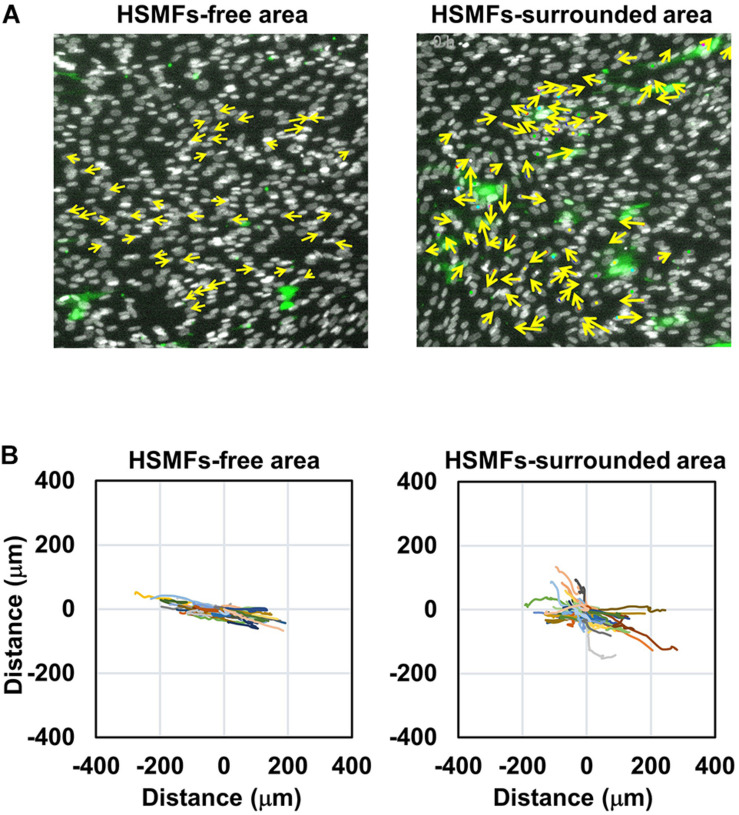
Dynamic behavior of HSMM cell migration in the monolayer comprising a small proportion of HSMF population. **(A)** A HSMM cell monolayer comprising 2% HSMF was prepared to analyze the migration of HSMMs in the HSMF-free and HSMF-surrounded areas. The HSMFs were stained with CellTracker Green^TM^ to distinguish them from HSMMs. All cells in the monolayer were stained with Hoechst 33342. The images were captured every 1 h during 48–72 h. Yellow arrow defines the direction of cell migration in the local area. **(B)** Representative trajectory plots showing directional migration of 100 HSMMs from each area. The direction and velocity of cell migration of 100 cells were measured at every position and plotted in the trajectory plot.

Next, the ability of HSMF in the HSMM monolayer to disrupt myoblast-to-myoblast contact was analyzed. Additionally, time-lapse analysis was performed to analyze the interaction and migration of HSMM and HSMF in the monolayer. The migration of HSMFs in the monolayer was hypothesized to disrupt the myoblast-to-myoblast contact, which may alter the directional migration and alignment of HSMMs. To verify this hypothesis, an HSMM monolayer containing CellTracker Green^TM^-labeled HSMFs of 2% (HSMM:HSMF ratio of 1:49) was prepared to observe the individual HSMF cell migration behaviors. The nuclei of cells in the monolayer were stained with Hoechst 33342, and the cell migration was tracked during 48–72 h. Directional migration of HSMMs in the HSMF-free area was compared with that of HSMM in the HSMF-surrounded area (Figure 3A). The trajectory plot revealed that HSMMs in the HSMF-surrounded area exhibited random migration, whereas those in the HSMF-free area exhibited unidirectional migration (Figure 3B and Supplementary Movie 1). This indicated that HSMFs are involved in myoblast-to-myoblast contact disruption.

### Quantification of Cell Migration and Alignment in Monolayer Comprising Various HSMF Proportions

**FIGURE 4:**
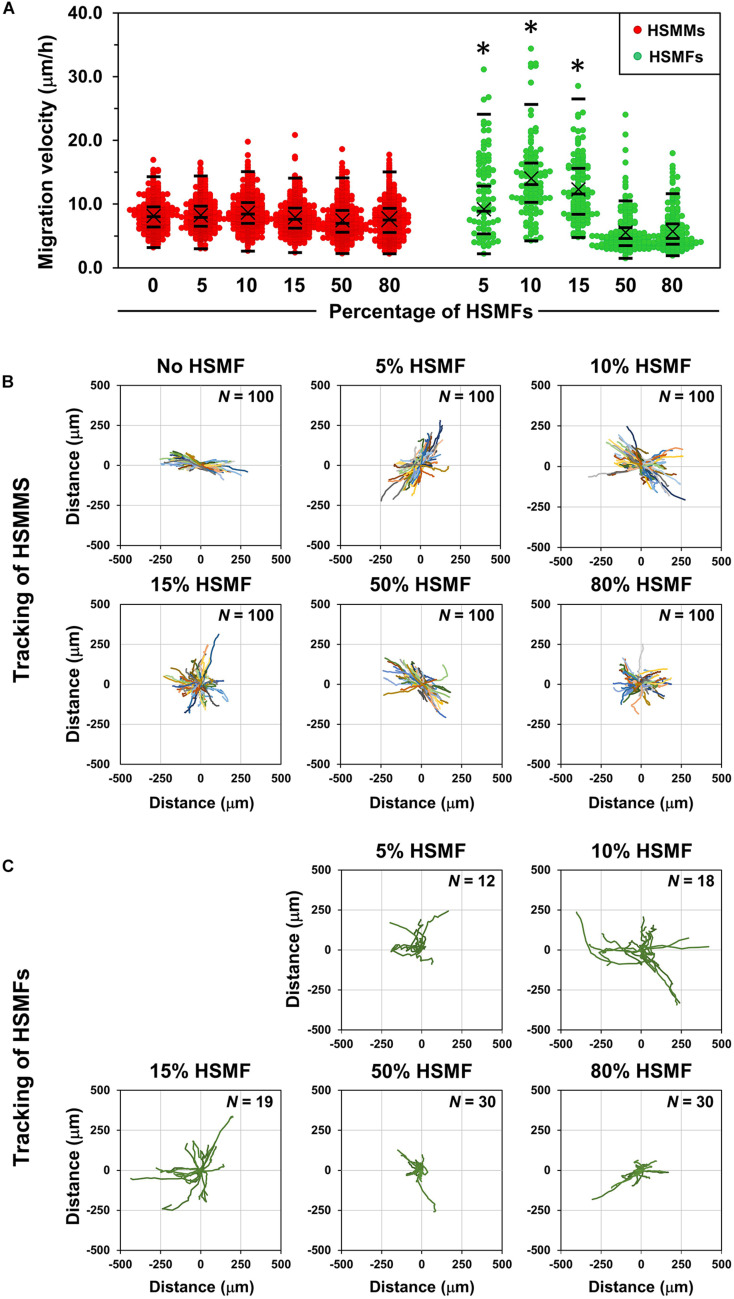
Migration behaviors of HSMMs and human skeletal muscle fibroblasts (HSMFs) in a monolayer sheet comprising various proportion of HSMF. **(A)** Various proportions of HSMFs, which were stained with CellTracker Green^TM^, were co-cultured with HSMM monolayer at an initial seeding density (*X*_0_) of 3.5 × 10^5^ cells/cm^2^. All cells in the monolayer were stained with Hoechst 33342 before observation. The images were captured every 1 h, and the position of cells was used to determine the migration velocity of HSMFs and HSMMs. The migration velocity of HSMFs in the monolayer derived from skeletal cells with varying proportion of HSMFs (5 to 15% HSMF) was higher than that in the monolayer derived from skeletal cells with HSMFs of more than 15%. The migration velocity of HSMM was unaffected in the presence of HSMFs. **(B)** The trajectory plot represents HSMM migration direction and distance in the monolayer comprising various proportions of HSMFs during 48–72 h of incubation. Representative images show data obtained from 100 HSMMs from each local area. **(C)** Trajectory plot represents the migration of HSMFs in monolayer comprising various proportions of HSMFs during 48–72 h of incubation. *P < 0.01; one-way analysis of variance (ANOVA), followed by Least-Significant Different (LSD) *post hoc* test.

**FIGURE 5:**
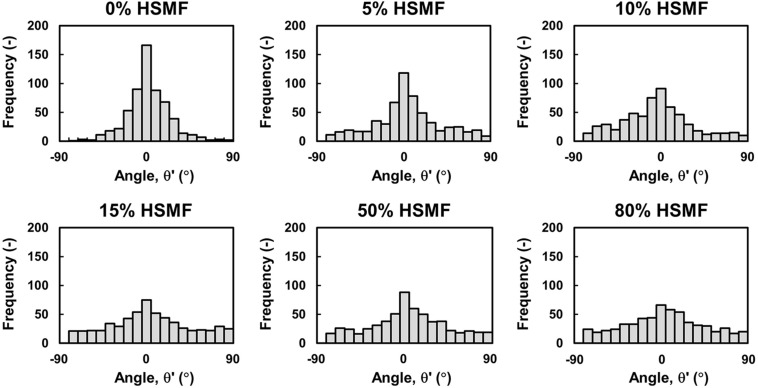
Quantitative analysis of HSMM alignment at high density in the monolayer comprising various proportions of HSMFs. Various proportions of HSMFs, which were stained with CellTracker Green^TM^, were co-cultured with HSMMs at an initial seeding density (*X*_0_) of 3.5 × 10^5^ cells/cm^2^. All cells in the monolayer were stained with Hoechst 33342 before observation. The positions (X,Y) of the cell at the initial (*t* = 0 h) and ending timepoints (*t* = 24 h) were used to calculate the migration angle (θ′) and were plotted in a histogram. Data were obtained from 100 HSMMs in each area. Six random areas from duplicated samples were analyzed (*n* = 600).

To confirm the role of HSMFs in myoblast-to-myoblast contact disruption, cell alignment in the monolayer prepared using HSMMs and various proportions of HSMFs was analyzed using an inverted microscope. The HSMM monolayer without HSMFs exhibited uniform cell alignment. The cell alignment was poor in some areas of the monolayer comprising a small proportion of HSMFs. The cell alignment was completely dysregulated in the monolayer comprising high proportion of HSMFs (Supplementary Figure 2). The HSMFs exhibited active migration in the monolayer comprising low proportions of HSMF (5 to 15% HSMF). Additionally, the migration velocity of HSMFs was significantly higher than that of HSMMs (*P* < 0.01). The migration velocity of HSMFs decreased when the proportion of HSMFs increased in the monolayer (Figure 4A). The trajectory plot of HSMM migration in the monolayer lacking HSMFs revealed unidirectional migration. The number of HSMMs exhibiting multidirectional migration increased with an increase in HSMF proportion (Figure 4B and Supplementary Movie 2). The HSMFs in the monolayer derived from skeletal muscle cells with 5, 10, and 15% HSMF exhibited active multidirectional migration (Figure 4C and Supplementary Movie 2). For quantitative analysis of cell alignment, the nucleus of HSMMs from three areas per sample was tracked to calculate the migration angle (θ’) as a robust metric to judge overall alignment (Figure 1A). The θ’ of HSMM migration in the monolayer comprising different HSMF proportions was plotted in a histogram (Figure 5). The degree of cell alignment disruption was determined by comparatively analyzing the standard deviation (σ) values, which indicate variation of directional migration (Supplementary Figure 3). Higher σ values indicate an increase in the myoblast alignment disruption level. The σ value of HSMM monolayer was 25.67 ± 4.67. The σ values of HSMFs in the monolayer derived from skeletal muscle cells with HSMF proportion of 5, 10, 15, 50 and 80% were 31.19 ± 9.52, 37.57 ± 3.27, 44.41 ± 5.70 (maximum σ value), 42.30 ± 3.43 and 43.73 ± 2.42, respectively. The staining of F-actin was performed to confirm the alignment disorder in HSMM monolayer containing 15% HSMF (Supplementary Figure 6). These results indicate that the HSMFs inhibit the myoblast-to-myoblast contact and consequently disrupt the myoblast alignment.

### Effect of HSMFs on GFP-HUVECs Network Formation in the Multilayered HSMM Sheet

**FIGURE 6:**
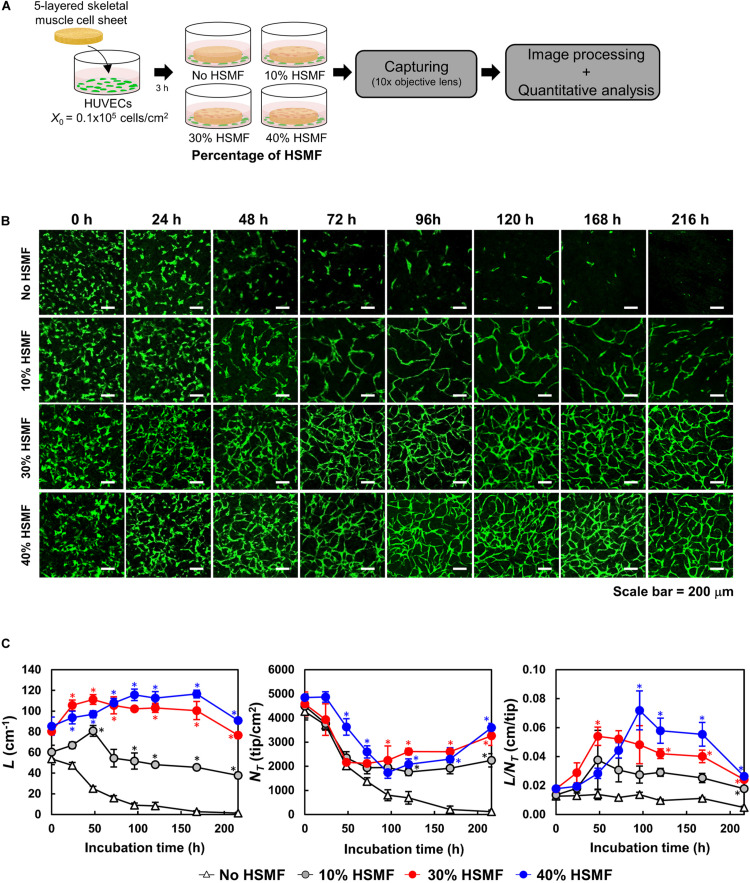
Time course of human umbilical vein endothelial cell (HUVEC) network formation in the five-layered HSMM sheet comprising various proportions of human skeletal muscle fibroblast (HSMF). **(A)** Five-layered HSMM sheets with various proportions of HSMFs were prepared and co-cultured with green fluorescent protein-tagged HUVECs (GFP-HUVECs) and observed for 216 h. **(B)** Representative images of HUVEC morphology. Scale bar: 200 μm. **(C)** Evaluation of HUVEC network formation with image processing software. *L*, total length (cm^–1^); *N*_*T*_, total tip number (tip/cm^2^); *L*/*N*_*T*_, extent of network formation (cm/tip). The bars show the standard deviation (SD) (*n* = 3). *P < 0.01; one-way analysis of variance (ANOVA), followed by Bonferroni *post hoc* test.

The efficiency of HUVEC network formation in the HSMM sheets comprising various proportions of HSMF was evaluated using an *in vitro* angiogenesis assay mimicking the transplantation area *in vivo* (Nagamori et al., 2013). The five-layered sheet was prepared using skeletal muscle cells with 0, 10, 30, and 40% HSMF. The sheet was then co-cultured with GFP-HUVECs (Figure 6A). Fabricated HSMM sheets morphology prepared by this method is shown in Supplementary Figure 4A. At *t* = 0 h, HUVECs were initially localized at the bottom of the HSMM sheet and vertically migrate into the inner portion of the sheet to generate a flat network inside the sheet after several days co-incubation (Supplementary Figure 4B). The network formation of HUVECs in the five-layered HSMM sheets was analyzed for 216 h based on the *L*, *N*_*T*_, and *L*/*N*_*T*_ parameters. The *X*_0_ value of HUVECs was 0.16 ± 0.01 × 10^5^ cells/cm^2^. As shown in Figure 6B, the GFP-HUVECs exhibited single and round shapes at the beginning of the incubation period (*t* = 0) However, early elongation of some GFP-HUVECs was observed in the sheets prepared using skeletal cells with HSMF proportions of 30 and 40%. Figure 6C shows the quantitative analysis of *L*, *N*_*T*_, and *L*/*N*_*T*_. At 24 h, the HUVECs exhibited elongation and were connected with each other in the HSMM sheets containing HSMFs. In contrast, the HUVECs exhibited poor connection with each other in HSMM sheets without HSMFs. The increase in elongation and connection resulted in increased *L* and decreased *N*_*T*_. At 48 h, early maturation of GFP-HUVECs was observed in the sheets prepared from skeletal cells with 30% HSMF. The maximum *L* (111.3 ± 4.71 cm^–1^) and *L*/*N*_*T*_ values (0.05 ± 0.01 cm/tip) were observed at 48 h, which were higher than those observed at 24 h and other conditions. The sheet prepared from skeletal cells with 10% HSMF exhibited a maximum *L*/*N*_*T*_ value of 0.034 ± 0.02 cm/tip, which was higher than the value at other time points of this condition. However, the elongation and smooth connection of GFP-HUVECs in sheet prepared from skeletal muscle cells with 40% HSMF, while the *L* and *L*/*N*_*T*_ values increased to a maximum of 115.76 ± 5.59 cm^–1^ and 0.07 ± 0.01 cm/tip, respectively at 96 h although *L*/*N*_*T*_ decreased in other conditions. This suggested late maturation of GFP-HUVEC network and indicated that different proportions of HSMFs in HSMM affect angiogenesis after transplantation in an *in vitro* angiogenesis assay.

## Discussion

The use of skeletal muscle myoblasts for myocardial transplantation was first demonstrated by Menasche et al. (2003). Since then, several studies have reported safety, feasibility, and improved heart performance with skeletal muscle myoblast transplantation (Siminiak et al., 2004; Dib et al., 2005a; Gavira et al., 2006). Besides, the myoblasts are prepared as cell sheets, therefore, enhancing the cell transplant efficiency and induction of therapeutic potential (Hata et al., 2006). Skeletal muscle myoblast sheet transplantation has been evaluated for the safety and therapeutic efficiency, and showed no serious arrhythmia or changes in the frequency of ventricular extrasystole frequency (Sawa et al., 2015). Although the myoblast sheets have demonstrated their therapeutic effects by producing various paracrine factors (Pouzet et al., 2001), the effect of skeletal muscle cells comprising different proportions of myoblasts and fibroblasts on cytokine production and angiogenesis has not been elucidated. In the present study, we addressed the regulation of cytokine production by specific cell types in the skeletal muscle cell sheets. The primary human skeletal muscle cells comprise 68.1% of HSMMs and 31.9% of HSMFs as shown in Supplementary Figure 1, were sorted based on CD56 expression, which is a myogenic marker, on the cell surface. The purity of sorted populations was determined by immunostaining of desmin and TE-7. The monolayer was prepared using various densities (mono-culture) and proportions (co-culture) of HSMMs and HSMFs. VEGF production was observed in the mono-cultures of HSMMs and HSMFs, with low productivity. However, the presence of a small proportion of HSMF in the HSMM monolayer resulted in enhanced VEGF productivity with maximum productivity observed in the monolayer of skeletal cells with 15% HSMF (Figure 2B). The production of HGF was observed only in the HSMF mono-culture (Figure 2C), whereas that of FGF2 was almost undetectable in the mono-cultures of both cell types (data not shown). Previous studies on primary human retinal pigment epithelial (RPE) cells have demonstrated that the expression of VEGF is highly upregulated at the edges of the scratched RPE layers after the physical disruption of RPE cell-to-cell interactions. This enhanced VEGF expression was correlated to delocalization of ZO-1, an important molecule for intercellular signal transduction in cells (Farjood and Vargis, 2017). Therefore, this study examined the role of cell-to-cell contact disruption in increasing the productivity of VEGF in the monolayers derived from HSMM and HSMF co-culture.

Then, the degree of cell-to-cell contact was evaluated by culturing the purified HSMMs and HSMFs at various *X*_0_ values. At high *X*_0_ value (3.5 × 10^5^ cells/cm^2^), the cells were in contact with the neighboring cells. Conversely, at low *X*_0_ value (0.1 × 10^5^ cells/cm^2^), the cells exhibited decreased contact with the neighboring cells. This study demonstrated that VEGF productivity in the HSMM monolayer was inversely proportional to the cell density (Figure 2D). However, the productivity of other cytokines in the HSMM monolayer was not associated with cell density. Additionally, the productivity of cytokines in the HSMFs was not dependent on the cell density (Figures 2D,E). Therefore, these data suggest that myoblast-to-myoblast contact can regulate VEGF production in HSMMs. Next, the effect of HSMF in the HSMM monolayer on myoblast-to-myoblast contact disruption was evaluated using cell tracking and time-lapse analysis to examine the migration behaviors. The free HSMFs actively migrated and affected the HSMM directional migration without affecting the migration rate of HSMMs (Figures 3, 4A and Supplementary Movie 1). HSMF migration in the monolayer was hypothesized to disrupt myoblast-myoblast contact, which alters the directional migration and alignment of HSMMs. The role of HSMF in HSMM alignment was evaluated by measuring the directional angle of cell migration, which was expressed as the CAI value, in the HSMM monolayers comprising various HSMF ratios. At low proportion in the HSMM monolayer, HSMFs exhibited active multidirectional migration. However, HSMFs exhibited inactive migration and aggregation at high proportion in the monolayer of skeletal cells with 50 and 80% HSMF (Figure 4C). In the pure HSMM monolayer, the HSMMs exhibited unidirectional migration. However, the presence of HSMFs increased the multidirectional migration of HSMMs (Figure 3B). In addition, the alignment disorder in monolayer containing low proportion of HSMF was confirmed by F-actin staining as shown in Supplementary Figure 6. Thus, the CAI values in the co-culture monolayer were higher than those in the mono-culture HSMM monolayer (Figure 5). These results support the hypothesis that the fibroblasts, which increase VEGF productivity in the HSMM monolayer, exhibit active migration and consequently disrupt the myoblast-to-myoblast contact. These findings indicate that HSMFs regulate the secretion of cytokines and that cytokine productivity is dependent on the proportion of HSMFs in the skeletal muscle sheets.

The effect of HSMFs in the skeletal muscle sheets on transplantation efficiency was evaluated using an *in vitro* angiogenesis assay. Five-layered HSMM sheets comprising various proportions of HSMFs were prepared and co-cultured with GFP-HUVECs (Figure 6A). The GFP-HUVECs in the HSMM sheet without HSMF exhibited elongation at an early time point but failed to connect and form a network. The presence of HSMFs enhanced the HUVEC network formation in HSMM sheets. However, the growth of GFP-HUVEC network was dependent on the proportion of HSMF and the levels of VEGF and HGF (Figures 6B,C). Various cytokines are involved in inducing angiogenesis. A previous study demonstrated that VEGF can increase blood vessel growth. However, the vessel was fragile and exhibited leakage. The combination of VEGF and HGF was reported to promote blood vessel growth and improve vessel integrity (Saif et al., 2010). In this study, the HUVECs initially exhibited network formation in the sheets prepared from skeletal cells with 10% HSMF, which markedly decreased over time. In contrast, the GFP-HUVECs in the sheets prepared from skeletal cells with 30 and 40% HSMF exhibited higher connectivity than those in sheets prepared from skeletal cells with 10% HSMF. The level of HGF under these conditions may be sufficient to promote strong connection among HUVECs when the VEGF productivity was similar. Moreover, HGF and VEGF levels may affect the time of network maturation. The maturation of HUVEC network in the sheet prepared from skeletal cells with 30% HSMF was observed at 48 h with maximum *L*/*N*_*T*_ value, whereas that in the sheets prepared from skeletal cells with 40% HSMF was observed at 96 h. The HUVEC network at 96 h was more stable than that at 48 h. The late maturation of the HUVEC network maybe due to low VEGF productivity and the stability of the network maybe due to high productivity of HGF.

The cytokine productivity was also measured from cultured media of five-layered HSMM sheets containing various proportion of HSMF co-incubated with GFP-HUVECs. The VEGF productivity in five-layered HSMM sheets containing 10% HSMF was three-fold greater than that of five-layered HSMM sheets without HSMF (Supplementary Figure 5A). The highest HGF productivity was observed in five-layered HSMM sheets containing 40% HSMF (Supplementary Figure 5B). Although levels of the VEGF and HGF increase in serum of myocardial infarction patients (Kubota et al., 2004; Seko et al., 2004; Atluri and Woo, 2008; Huang et al., 2020), it is noteworthy that the amount of these cytokines secreted from the five-layered HSMM sheets co-cultured with HSMFs were much higher, suggesting the ability of the five-layered HSMM sheets in induction of angiogenesis toward the injure site.

This study did not evaluate the effect of ECM protein deposition on HSMM sheets comprising various ratios of HSMFs. Hence, the effects of ECM in promoting HUVEC network formation and supporting network stability cannot be excluded. In this study, the sheets prepared from skeletal cells with HSMF higher than 50% were not comparatively analyzed. The sheets containing a high proportion of HSMFs were fragile and self-contracted during preparation, which can be attributed to the aggregation of HSMFs at high proportions (Figures 4A,C and Supplementary Movie 2) due to the strong connection between HSMFs.

## Conclusion

**FIGURE 7:**
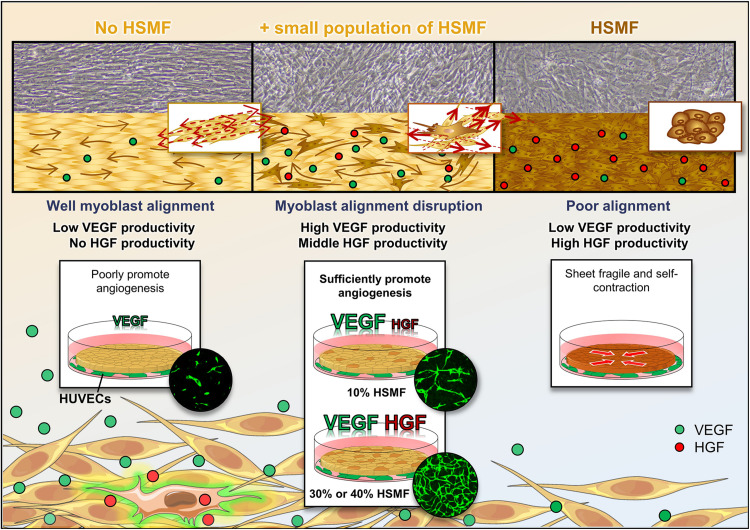
Schematic representation of the effect of human skeletal muscle fibroblasts (HSMFs) co-cultured in skeletal muscle cell sheets on cytokine balance and angiogenesis.

This study demonstrated that different ratios of HSMM and HSMF differentially affect cytokine balance and angiogenesis as showed in Figure 7. Fibroblasts secrete high levels of HGF and promote VEGF production in HSMM to maintain cytokine balance, which is potentially mediated through inhibition of active HSMM migration that results in the loss of myoblast-to-myoblast contact. The co-culture of HSMF and HSMM at a suitable ratio promotes angiogenesis and cytokine production. The findings of this study can be used to improve the efficiency of HSMM sheets or engineered tissue for transplantation and highlights the role of fibroblasts in VEGF secretion regulation from adjacent tissue.

## Data Availability Statement

The raw data supporting the conclusions of this article will be made available by the authors, without undue reservation.

## Author Contributions

PT performed the experiments, data analysis and interpretation, and drafted and finally edited manuscript. MK significantly contributed to revise the manuscript and supervised the project. All authors designed the experiments and discussed the results and approved the final version of the manuscript.

## Conflict of Interest

The authors declare that the research was conducted in the absence of any commercial or financial relationships that could be construed as a potential conflict of interest.
