# Supplementary material for: Effect of Co-culturing Fibroblasts in Human Skeletal Muscle Cell Sheet on Angiogenic Cytokine Balance and Angiogenesis
Source: Front Bioeng Biotechnol. 2020 Sep 23;8:578140. doi: 10.3389/fbioe.2020.578140 (PMC7542332; doi:10.3389/fbioe.2020.578140)
Supplement: Supplementary file 3 [file Data_Sheet_1.pdf]

Supplementary Material

1 Supplementary Data (Movies)

Movie 1. Time-lapse analysis of human skeletal muscle myoblast (HSMM) migration in the monolayer prepared from skeletal cells with 2% human skeletal muscle fibroblast (HSMF) during 48-72 h of incubation. The HSMFs were stained with CellTracker Green^{TM} to distinguish them from HSMMs. All the cells in the monolayer were stained with Hoechst 33342. The images were captured every 1 h and the migration of 100 HSMMs each from the free-HSMF area and the HSMF-surrounded area was tracked.

Movie 2. Time-lapse analysis of directional migration of human skeletal muscle myoblast (HSMMs) in the monolayer comprising various proportions of human skeletal muscle fibroblasts (HSMFs) during 48-72 h of incubation. Various proportions of HSMFs, which were stained with CellTracker Green^{TM}, were co-cultured with HSMM at an initial seeding density (X_0) of 3.5×10^5 cells/cm^2. All cells in the monolayer were stained with Hoechst 33342 before observation. The images were captured every 1 h for 24 h. In each area, 100 HSMMs were tracked.

2 Supplementary Figures

A

B

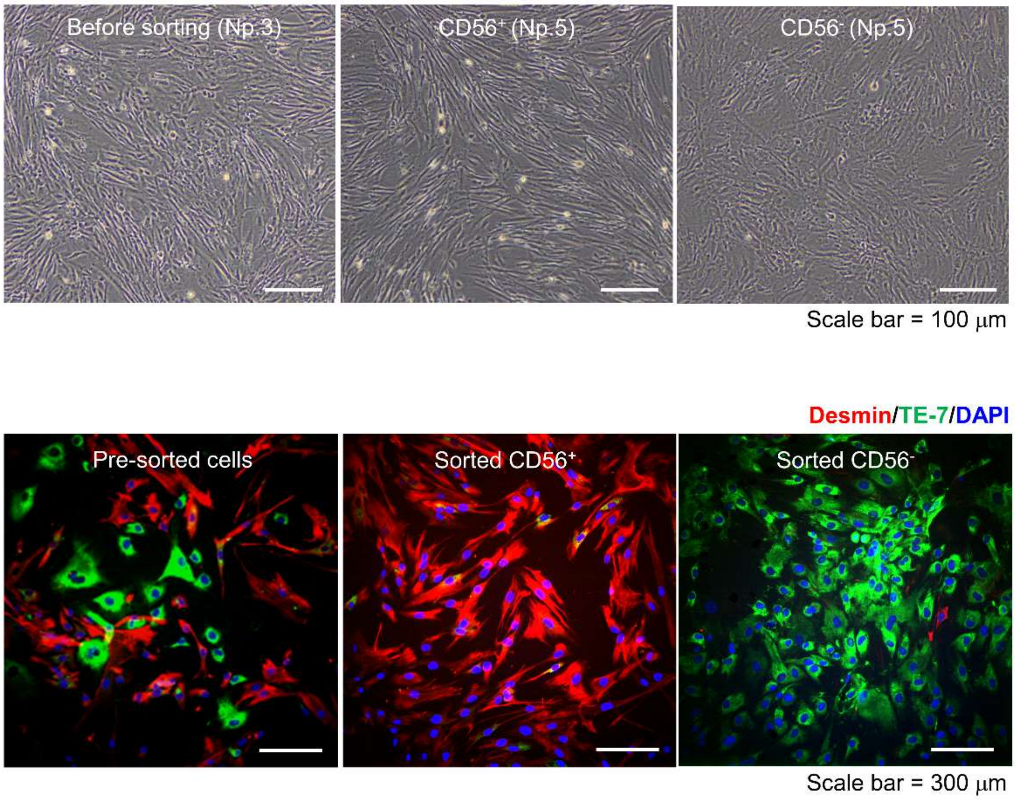

C

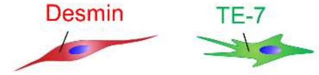

	Myogenic cell	Fibroblast
Before sorting	68.1%	31.9%
Sort CD56+	98.4%	1.6%
Sort CD56-	5.0%	95.0%

D

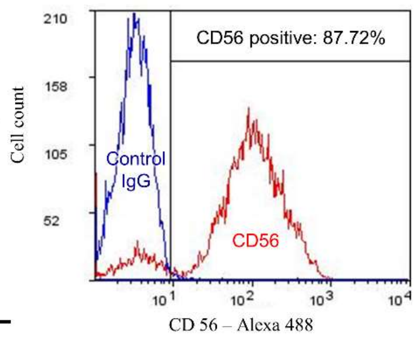

Supplement figure 1. Characterization and quantification of cells during the culturing and sorting processes. (A) Characteristics of presorted and post-sorted cells (CD56+ and CD56− cells) cultured in SkGM-2 media were analyzed under an inverted microscope. Scale bar, 100 µm. Immunostaining of the presorted cells after 5 days of culturing revealed a mixed population of myogenic cells (desmin+) and fibroblasts (desmin−, TE-7+). (B-C) After sorting, the CD56+ cells mainly constituted myogenic

cells (desmin+), whereas the CD56− cells mainly constituted fibroblasts. Scale bar, 200 μm. Flow cytometry analysis of presorted human skeletal muscle myoblasts (HSMM) revealed a mixed population of CD56+ and CD56− cells when compared with the negative control (IgG isotype) (D).

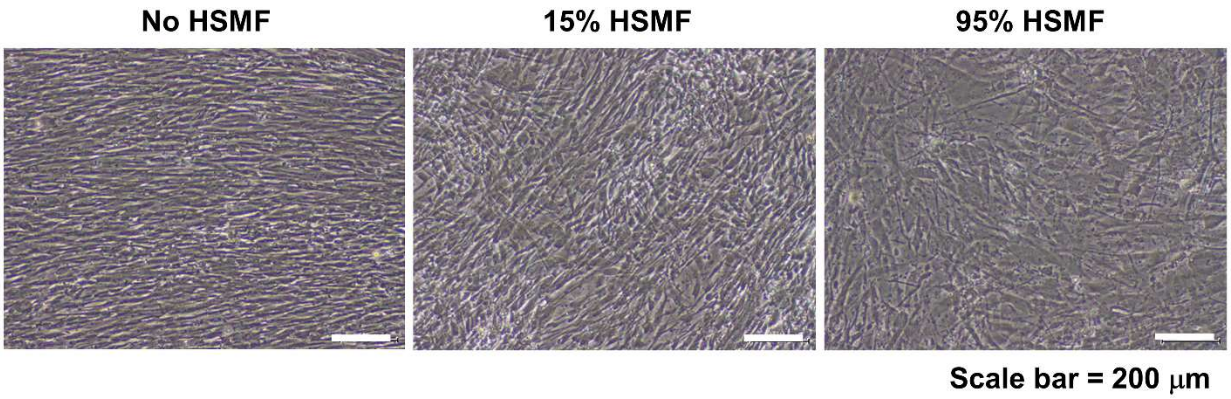

Supplement figure 2. Analysis of cell alignment in the monolayer. The monolayers prepared using skeletal cells with various proportions of human skeletal muscle fibroblast (HSMF) and human skeletal muscle myoblast (HSMM) (0, 15 and 95% HSMF) were analyzed under a 10X objective lens of an inverted microscope.

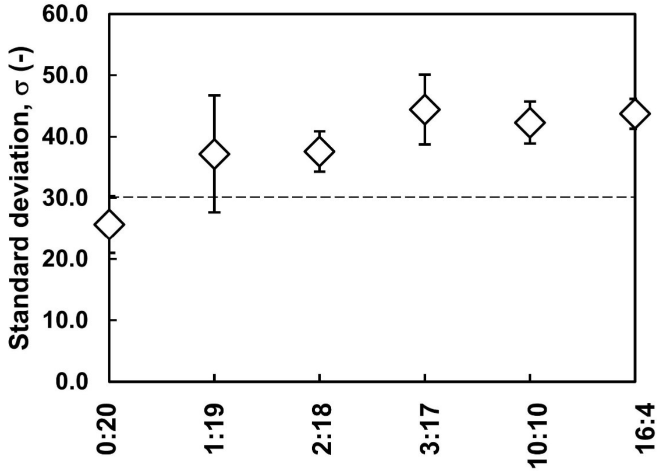

Supplement figure 3. Quantitative analysis of myoblast alignment disruption. Cell alignment was quantitated by measuring the direction angle of cell migration. Data were obtained from 600 human skeletal muscle myoblasts (HSMMs) in duplicates. The standard deviation (σ) values were used as a metric of variation index of direction migration and were compared among different conditions. A high σ value indicated multidirectional migration and high myoblast alignment disruption.

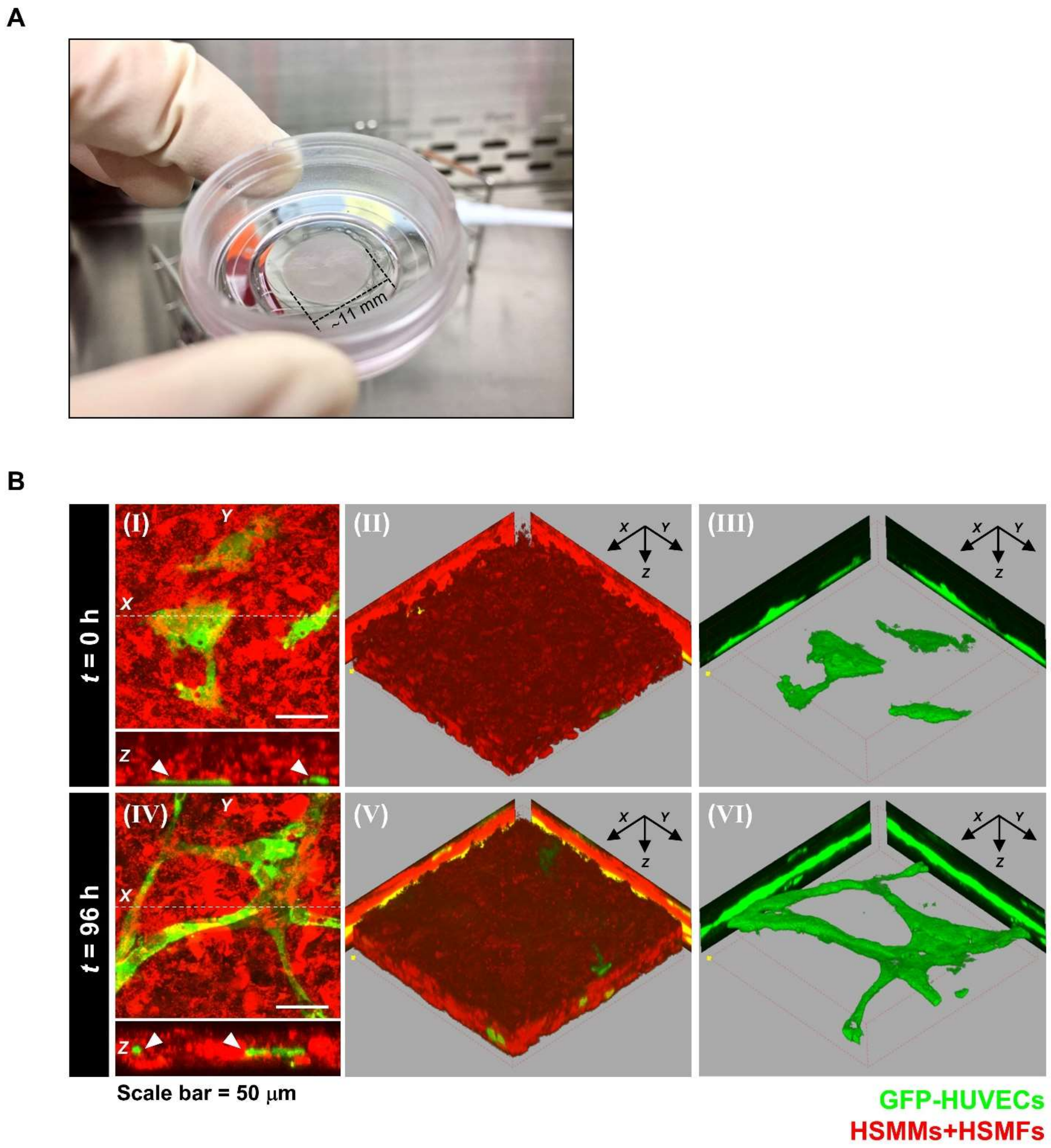

Supplement figure 4. Characterization of 5-layered human skeletal muscle cell sheet co-cultured with human umbilical vein endothelial cells (HUVECs). (A) Representative image shows morphology of multilayered HSMM sheet after transferring to the center of a 35 mm culture dish, which was seeded with GFP-HUVECs (X_0 of 0.1×10^5 cells/cm^2) in EGM-2 at 37°C and 5 % CO_2 for 24 h. (B) The stacking images were captured at $t = 0$ h (I, II and III) and $t = 96$ h (IV, V and VI) by confocal laser scanning microscope using 60X objective lens. The 3D images were generated to show the 3D structure of cell sheet and HUVECs network formation inside.

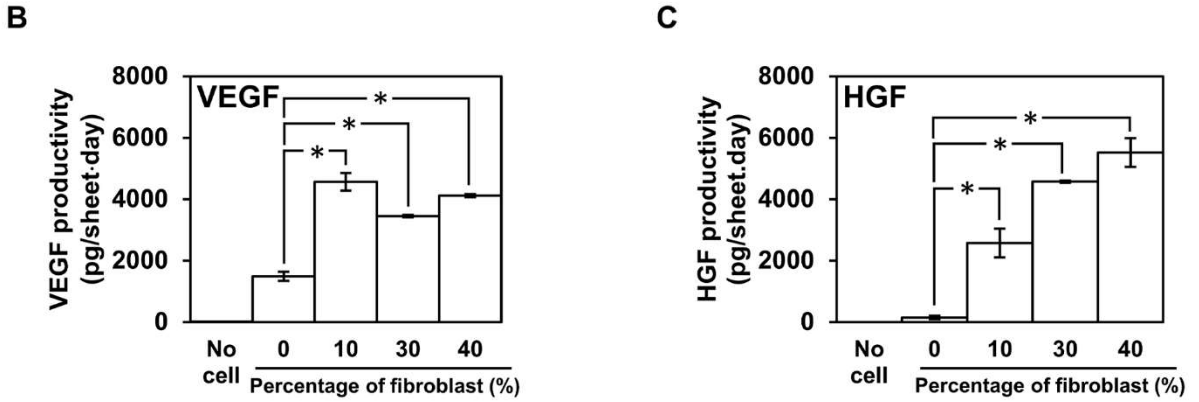

Supplement figure 5. Cytokine productivity in 5-layered human skeletal muscle myoblast (HSMM) sheet containing various proportion of human skeletal fibroblast (HSMM) co-cultured with GFP-HUVECs at 48 h. (A) Effect of co-culturing HSMFs and HSMMs with various proportions in 5-layered HSMM sheets on vascular endothelial growth factor (VEGF) productivity and (B) hepatocyte growth factor (HGF) productivity. Data are represented as average cytokine productivity ± standard deviation from triplicate samples (n = 3). *P < 0.01; one-way analysis of variance (ANOVA), followed by Bonferroni post-hoc test.

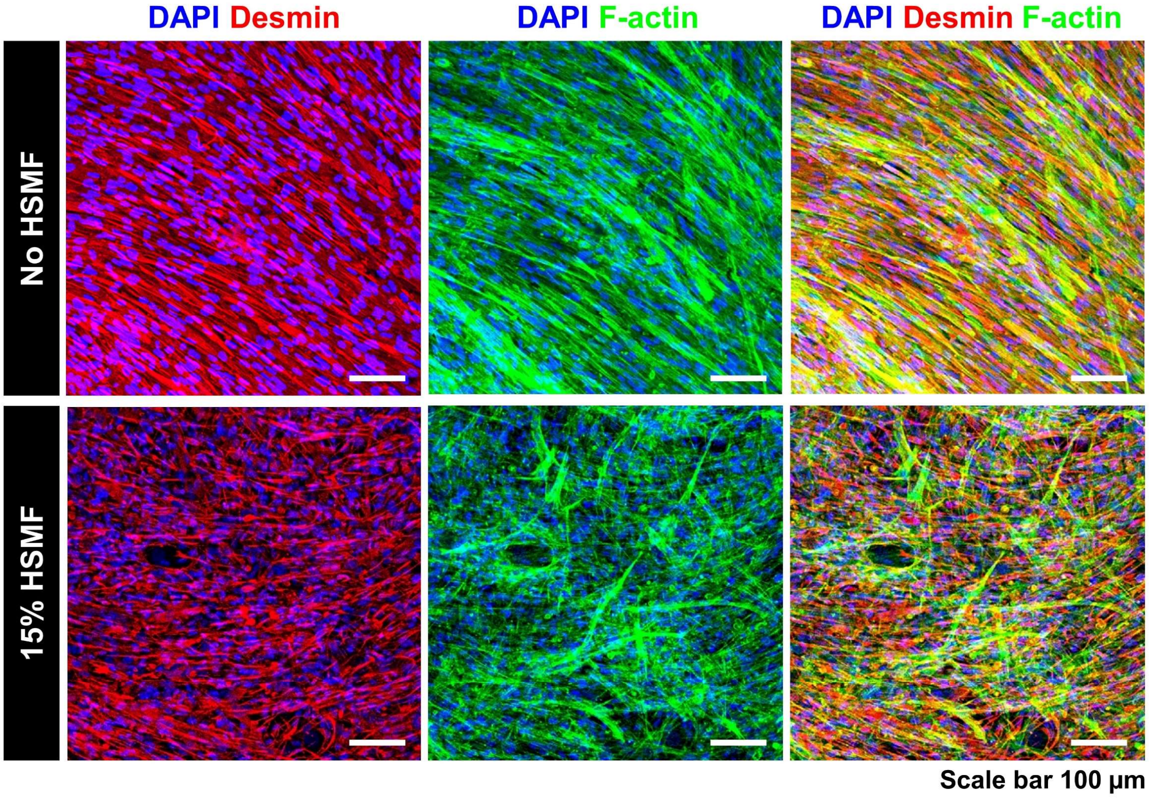

Supplement figure 6. Fluorescent staining images of desmin (red) and F-actin (green) structures towards nuclei (blue) in human skeletal muscle myoblast (HSMM) monolayers containing with or without 15% human skeletal muscle fibroblast (HSMF).

3 Material and method

3.1 Fluorescent staining

For monolayer immunostaining, the samples were washed twice with phosphate buffered saline (PBS) before fixing with 4% paraformaldehyde in PBS (Wako Pure Chemical Industries, Tokyo, Japan) for 15 min at room temperature. Then, the monolayers were permeabilized with 0.5% triton X-100 in PBS for 20 min. Nonspecific proteins were then blocked for 90 min with Block Ace (Dainippon Sumitomo Pharma Co., Ltd., Osaka, Japan) at room temperature. After that, the cells were incubated with anti-desmin antibody (Y66) (Cat. No. ab32362, Abcam, USA) at 1:250 dilution prepared in deionized water containing 10% Block Ace at 4°C overnight. Then, the samples were washed twice with Tris-buffered saline (TBS) and immunolabeled with Alexa Fluor® 594 goat anti-rabbit IgG (Cat. No. A11001, Molecular Probes, Life Technologies, USA) at 1:250 dilution prepared in deionized water containing 10% Block Ace at room temperature for 1 h. The F-actin and nuclei were fluorescent stained with Alexa Fluor™ 633 Phalloidin (Cat. No. A22284, Molecular Probes, Life Technologies, USA) and 4¢,6-diamidino2-phenylindole (Cat. No. D1306, Molecular Probes, Life Technologies, USA), respectively. The stained samples were observed by a confocal laser scanning microscope (FV-1000; Olympus, Tokyo, Japan).
